# Glances at the Hospitals

**Published:** 1905-12-23

**Authors:** 


### GLANCES AT THE HOSPITALS.

### ROYAL VICTORIA HOSPITAL, BELFAST.

The total receipts were £11,582, and the expenditure was £10,509. The workpeople's subscriptions amounted to the respectable sum of £3,477. At the beginning of the year 160 patients remained in the wards, and 2,298 new cases were admitted during the year, making a total of 2,458 in-patients. Of the new cases 888 were medical, and 1,410 were surgical. There were 146 deaths, but 27 patients were admitted in a dying state. Excluding these the average mortality was 5.1 per cent. The average daily number of patients in residence was 158, and the duration of each patient's stay in the wards was twenty-six days. The total cost per head per day was 2s. 8½d. The medical and surgical tables show the numbers under treatment for various diseases, and there is a column showing the number of deaths. There were 43 cases of bronchitis with 1 death;

25 cases of pneumonia with 2 deaths; 25 cases of diphtheria with 3 deaths. Among many other operations appendicectomy was performed 21 times without a death. There were 29 amputations (12 being of the leg and 4 of the thigh) without a death. Of 12 cases of ovariotomy all were successful. The total number of operations was 886, and the number of deaths subsequent to operation was 27. We were unable to find an anæsthetic table.

